# Case of Apoplectic Chill, Acute Rheumatism, and Paralysis Combined

**Published:** 1847-03

**Authors:** J. E. Stewart

**Affiliations:** Jackson, Tennessee


### SELECTIONS

FROM

### AMERICAN AND FOREIGN JOURNALS.

---

*A Case of Apoplectic Chill, Acute Rheumatism, and Paralysis combined.* By J. E. Stewart, M. D., of Jackson, Tennessee.—W. W. Williams, ætat. 52 years; laborer, of robust habit and general good health, and had always been temperate, was attacked, July 25th, while in the woods chopping, with what both he and his son, who was with him, considered to be a chill. They immediately set out for home, and after resting several times reached the house. Immediately after his arrival he fell into a state of insensibility, and so remained for several hours; during which time I was sent for. On my arrival I found him in the following state, viz: rather comatose; the face flushed, and apparently swollen; breathing labored, but not stertorous; frequent sighing; when spoken to answers slowly, but correctly. When I took hold of his arm to examine his pulse, he shrunk from my touch and complained bitterly. I now ascertained that he had lost all command over the voluntary muscles of the right arm, and that the shoulder and elbow joints were greatly swollen, red, and exceedingly painful. So acute was the pain in the deltoid and other muscles of the arm, that he could not bear the weight and friction of his shirt sleeve.

℞. Venesection ℥xx., sul. mag. flo. sul. *āā* ʒ½., and on the following morning one of the following pills every two hours, quinine, gr.iv., potas. hyd., gr.ss.

July 27th. Has had no further paroxysm, pulse 90, rather hard; venesec. ℥xv., other treatment continued, to which was added a stimulating liniment.

28th. Bowels patulous; pulse 70, compressible; appetite good. Discontinue the purgatives and continue the other treatment.

29th. Symptoms same; has slept well; continue treatment.

30th. There being but little, if any amendment, in either the acute sensibility or motion of the arm, I determined to add to the above treatment the following: ℞, strychninæ gr.i., aquæ puræ ℥i. A teaspoonful to be taken morning, noon, and night.

31st. Symptoms in *statu quo;* treatment continued.

August 1st. Slightly better, can move his fingers; arm not quite so painful to the touch; bowels rather constipated; ordered two of the following pills at bed-time: ℞. ext. col. comp. ℈i., ant. tart. gr.iv., ol. carui. m. iii.; M. massa, divid. in pil. xx. Other treatment continued.

2d. Better; bowels have been operated on four or five times; pulse 75, soft. The above treatment was continued up to the latter part of August, by which time he had so far recovered as to be able to return to his ordinary occupation, to wit: that of farming.

Sept. 25th, 1846. Had another attack in all respects similar to the first, except that it affected the left arm, so far as loss of motion and pain, or rather soreness, was concerned, as the first paroxysm had done the right. There was no swelling of the left arm. Former treatment resumed.

26th. Bowels loose; pulse soft and compressible; appetite good.

27th. Symptoms the same; cannot move the left arm.

28th. Somewhat better. Continue treatment.

29th. Still mending; continue treatment.

30th. Convalescent.

Oct. 26th. Had another attack similar to the one already described, but affecting the right arm as at first. I now substituted spts. turp. m. 3 night and morning, for the hyd. pot.

27th. Better; treatment continued.

28th. Better; treatment continued.

Nov. 2d. Convalescent.

*Remarks.*—I believe you will agree with me that the above is a most singular and very interesting case. It was not genuine apoplexy, for the paroxysm was ushered in by a chill, and there was no stertor. It was not genuine paralysis attendant upon apoplexy; because, although motion was lost, yet, the sensibility was most acute, and besides, there were swelling and redness accompanying the first and third paroxysms, neither of which accompanies hemiplegia or general paralysis. Believing the disease to be a complication, as above headed, I combined my remedies accordingly, and with what success the above detail will show.—*Missouri Medical and Surgical Journal.*

